# Subacute puerperal uterine inversion secondary to a large uterine myoma: a case report

**DOI:** 10.1093/jscr/rjaf1016

**Published:** 2026-01-02

**Authors:** Juan Yang, Shilin Zhong, Wei Cheng, Yuqing Deng, Ying Wang, Yuzhen Liu

**Affiliations:** Guangdong Medical University, No. 2 Wenming East Road, Xiashan District, Zhanjiang, Guangdong 524023, China; Center of Obstetrics and Gynecology, Peking University Shenzhen Hospital, No. 1120 Lianhua Road, Futian District, Shenzhen, Guangdong 518036, China; Institute of Obstetrics and Gynecology, Shenzhen Peking University-The Hong Kong University of Science and Technology Medical Center, No. 1120 Lianhua Road, Futian District, Shenzhen, Guangdong 518036, China; Shenzhen Key Laboratory of Early Diagnosis Technology for Female Major Diseases, No. 1120 Lianhua Road, Futian District, Shenzhen, Guangdong 518036, China; Guangdong Medical University, No. 2 Wenming East Road, Xiashan District, Zhanjiang, Guangdong 524023, China; Center of Obstetrics and Gynecology, Peking University Shenzhen Hospital, No. 1120 Lianhua Road, Futian District, Shenzhen, Guangdong 518036, China; Institute of Obstetrics and Gynecology, Shenzhen Peking University-The Hong Kong University of Science and Technology Medical Center, No. 1120 Lianhua Road, Futian District, Shenzhen, Guangdong 518036, China; Shenzhen Key Laboratory of Early Diagnosis Technology for Female Major Diseases, No. 1120 Lianhua Road, Futian District, Shenzhen, Guangdong 518036, China; Center of Obstetrics and Gynecology, Peking University Shenzhen Hospital, No. 1120 Lianhua Road, Futian District, Shenzhen, Guangdong 518036, China; Institute of Obstetrics and Gynecology, Shenzhen Peking University-The Hong Kong University of Science and Technology Medical Center, No. 1120 Lianhua Road, Futian District, Shenzhen, Guangdong 518036, China; Shenzhen Key Laboratory of Early Diagnosis Technology for Female Major Diseases, No. 1120 Lianhua Road, Futian District, Shenzhen, Guangdong 518036, China; Center of Obstetrics and Gynecology, Peking University Shenzhen Hospital, No. 1120 Lianhua Road, Futian District, Shenzhen, Guangdong 518036, China; Institute of Obstetrics and Gynecology, Shenzhen Peking University-The Hong Kong University of Science and Technology Medical Center, No. 1120 Lianhua Road, Futian District, Shenzhen, Guangdong 518036, China; Shenzhen Key Laboratory of Early Diagnosis Technology for Female Major Diseases, No. 1120 Lianhua Road, Futian District, Shenzhen, Guangdong 518036, China; Center of Obstetrics and Gynecology, Peking University Shenzhen Hospital, No. 1120 Lianhua Road, Futian District, Shenzhen, Guangdong 518036, China; Institute of Obstetrics and Gynecology, Shenzhen Peking University-The Hong Kong University of Science and Technology Medical Center, No. 1120 Lianhua Road, Futian District, Shenzhen, Guangdong 518036, China; Shenzhen Key Laboratory of Early Diagnosis Technology for Female Major Diseases, No. 1120 Lianhua Road, Futian District, Shenzhen, Guangdong 518036, China; Center of Obstetrics and Gynecology, Peking University Shenzhen Hospital, No. 1120 Lianhua Road, Futian District, Shenzhen, Guangdong 518036, China; Institute of Obstetrics and Gynecology, Shenzhen Peking University-The Hong Kong University of Science and Technology Medical Center, No. 1120 Lianhua Road, Futian District, Shenzhen, Guangdong 518036, China; Shenzhen Key Laboratory of Early Diagnosis Technology for Female Major Diseases, No. 1120 Lianhua Road, Futian District, Shenzhen, Guangdong 518036, China

**Keywords:** uterine inversion, postpartum, leiomyoma, myomectomy, magnetic resonance imaging

## Abstract

Uterine inversion is a life-threatening obstetric emergency characterized by the turning inside out of the uterine fundus into the endometrial cavity or beyond the cervix. Common risk factors include fundal pressure, cord traction, placental disorders, and uterine atony. We report a rare case of subacute puerperal uterine inversion caused by a giant prolapsing submucosal leiomyoma in a 36-year-old woman who presented on postpartum day 19 with fever, abdominal pain, and prolapse of a vaginal mass. Imaging revealed a large submucosal myoma and grade I uterine inversion. Initial manual reduction failed. Successful management was achieved via laparotomy, with myomectomy through a uterine incision, uterine restoration, and intrauterine balloon tamponade.A large submucosal myoma can be a significant risk factor for late puerperal uterine inversion. Timely diagnosis using magnetic resonance imaging and individualized surgical intervention are crucial for successful anatomical correction and fertility preservation.

## Introduction

Uterine inversion, the collapse of the uterine fundus into the endometrial cavity, is classified as acute (<24 hours), subacute (24 hours to 30 days), or chronic (>30 days) [1]. Most cases occur during the immediate puerperium [2], with approximately 83.4% being acute [3]. It is a significant cause of traumatic shock, hemorrhagic shock, and puerperal infection. Common risk factors include fundal pressure, cord traction, and placental disorders [1]. The condition often has an insidious onset and atypical manifestations, leading to potential diagnostic delays. We report a rare case of subacute puerperal uterine inversion caused by a giant prolapsing submucosal leiomyoma to enhance clinical awareness and management.

## Case presentation

A 36-year-old gravida 3 para 1 woman, who had been diagnosed one year earlier with a right intramural uterine myoma (38 × 96 mm, ≥50% subserosal), presented at 39^+1^ weeks of gestation in her second pregnancy. Serial antenatal ultrasounds documented progressive enlargement of the myoma, which reached 111 × 89 × 92 mm prior to delivery. She underwent an uncomplicated spontaneous vaginal delivery of a healthy 3000 g infant, recovered smoothly in the immediate postpartum period, and was discharged on postpartum day 2.

On postpartum day 14, she developed a persistent high-grade fever refractory to antibiotics, with imaging showing myoma enlargement and leukocytosis. A CT scan on day 17 revealed ileocecal inflammation and uterine fluid. By postpartum day 19, she reported abdominal pain, lumbar soreness, prolapse of a vaginal mass, and vaginal bleeding (approximately 1.5 times her usual menstrual flow). The mass prolapsed further during the Valsalva maneuver, and she developed urinary retention requiring catheterization. Repeat ultrasound showed a 20 mm fundal serosal indentation and a 90 × 60 mm isoechoic mass on the right uterine wall. Laboratory studies yielded the following: WBC 10.54 × 10^9^/L, hemoglobin 101 g/L, procalcitonin 0.47 ng/mL, and IL-6 31 pg/mL. She was subsequently admitted.

**Figure 1:**
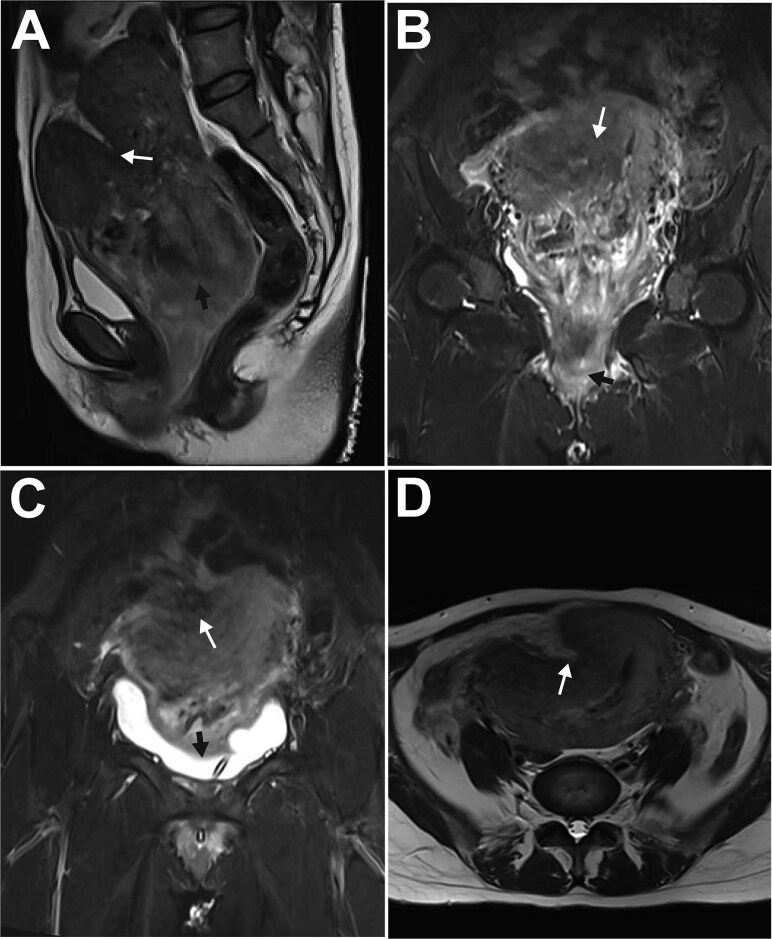
MRI features of subacute uterine inversion secondary to a large uterine myoma. A: Paramedian sagittal T2-weighted turbo spin-echo image showed a lobulated fundal depression (white arrow) and the underlying uterine myoma (black arrow); B: Paramedian sagittal T2-weighted turbo inversion recovery magnitude (TIRM) image demonstrated the myoma prolapsing from the uterine cavity into the vagina (white arrow), with a portion extending through the introitus (black arrow); C: Coronal T1-weighted image revealed the myometrial indentation (white arrow) and the compressed urinary bladder (black arrow); D: Axial T2-weighted turbo spin-echo image showed the myometrial depression (white arrow) on the right anterofundal wall.

On physical examination, she was afebrile (36.6°C) but tachycardic (117 bpm). Her pre-pregnancy BMI was 17.6 kg/m^2^ (height 165 cm, weight 48 kg). A dark red, tongue-shaped mass (3 × 3 × 5 cm) protruded from the vaginal introitus, with a larger firm mass (7 × 8 × 9 cm) palpable inside the vagina. The cervix was palpable around the mass; the uterus was anteverted, equivalent in size to a 12-week gestation, mobile, and non-tender. Pelvic magnetic resonance imaging (MRI) confirmed a fundal myometrial indentation, an 86 × 82 × 105 mm heterogeneous intrauterine mass (suggestive of a degenerated submucosal myoma), and a mass extending from the cervical os to the introitus (Fig. 1). Admission diagnoses were: 1) Subacute uterine inversion (Grade I); 2) Prolapsed large submucosal myoma; and 3) Puerperal infection.

Intravenous antibiotics were administered. The following day, ultrasound-guided excision of the tongue-shaped introital mass and manual reduction were attempted; however, the vaginal mass reprolapsed immediately after initial reduction. A subsequent attempt at reduction combined with cervical cerclage was unsuccessful, prompting laparotomy.

**Figure 2:**
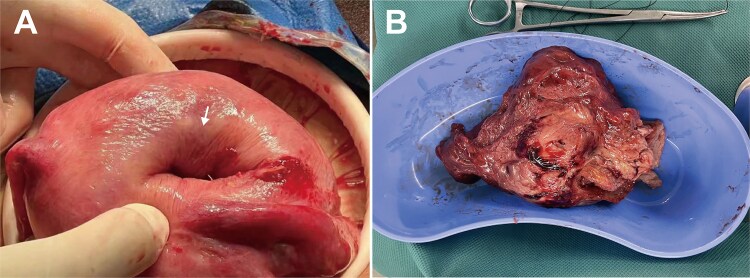
Intraoperative findings during laparotomy for uterine inversion. A: Enlarged uterus with smooth serosa; a 2.5 cm-diameter, 4 cm-deep distinct depression (white arrow) on the right anterofundal wall; B: Surgically enucleated, bisected uterine myoma, showing typical leiomyoma gross features: Dark red color, soft consistency, whorled cut surface.

At laparotomy, the uterus was sized at 12 weeks with a 2.5 × 4 cm depression on the right anterofundal wall (Fig. 2A). Following an unsuccessful transvaginal reduction, a uterine incision was made over the depression, exposing and enucleating a 9 cm submucosal myoma (Fig. 2B). The myoma bed was sutured, and minimal endometrial bleeding was controlled with intrauterine balloon tamponade alongside pelvic drain placement. Postoperative recovery with antibiotics, uterotonics, and anemia correction was uneventful. The balloon was removed the next day, and the patient was discharged on postoperative day 7. Pathology confirmed uterine leiomyoma with vascular changes in the main mass and infarction in the introital specimen. A follow-up ultrasound on day 15 was normal.

## Discussion

This case of subacute puerperal uterine inversion was triggered by a giant prolapsing submucosal leiomyoma. The inversion likely resulted from a combination of forceful traction and structural vulnerability: marked myoma enlargement created a sustained pull on the uterine wall, while the soft, postpartum myometrium (potentially weakened by infection and the patient's low BMI) offered diminished resistance. Multiparity and a triggering event like coughing may have further contributed.

This subacute presentation on postpartum day 19, marked by less severe hemorrhage possibly from partial endometrial repair, featured the characteristic triad of abdominal pain, mass prolapse, and urinary obstruction. MRI was crucial for diagnosis: it confirmed the inversion and delineated the myoma, demonstrating a "V-shaped" fundal indentation on sagittal view [4] and a focal "lobulated" appearance on axial view instead of the classic "bull's-eye" sign [1], which correlated with the surgical finding of a right anterofundal depression.

Management of puerperal uterine inversion depends on acuity and associated factors. Non-surgical reduction is first-line in acute cases [5]. However, a large myoma, as in this case, often precludes successful manual reduction, necessitating surgery. An abdominal approach is preferred in most cases [6]. For women not desiring future fertility, a transabdominal approach with myomectomy via the thinned, inverted uterine wall followed by hysterorrhaphy is effective. For those wishing to preserve fertility, a transverse lower uterine segment incision may be preferable for myoma removal and uterine restoration, potentially reducing future rupture risk. Concomitant cervical cerclage [7] or intrauterine balloon tamponade [8] can help prevent recurrence and control bleeding, especially with a patulous cervix. Preoperative planning should carefully assess inversion morphology, myoma characteristics, and their spatial relationship.

In conclusion, this case establishes a large submucosal myoma as a risk factor for late puerperal uterine inversion. Recognizing its distinct clinical and radiological features is crucial for timely diagnosis. Management must be individualized, focusing on anatomical correction, eradication of the myoma, and preservation of fertility.
